# Oxidative Stress and Inflammation: Essential Partners in Alcoholic Liver Disease

**DOI:** 10.1155/2012/853175

**Published:** 2012-03-01

**Authors:** Aditya Ambade, Pranoti Mandrekar

**Affiliations:** Department of Medicine, University of Massachusetts Medical School, 364 Plantation Street, Worcester, MA 01605, USA

## Abstract

Alcoholic liver disease (ALD) is a multifaceted disease that is characterized by hepatic steatosis or fat deposition and hepatitis or inflammation. Over the past decade, multiple lines of evidence have emerged on the mechanisms associated with ALD. The key mechanisms identified so far are sensitization to gut-derived endotoxin/lipopolysaccharide resulting in proinflammatory cytokine production and cellular stress due to oxidative processes, contributing to the development and progression of disease. While oxidative stress and inflammatory responses are studied independently in ALD, mechanisms linking these two processes play a major role in pathogenesis of disease. Here we review major players of oxidative stress and inflammation and highlight signaling intermediates regulated by oxidative stress that provokes proinflammatory responses in alcoholic liver disease.

## 1. Introduction

The pathogenesis of alcoholic liver disease (ALD) is a consequence of chronic alcohol abuse and approximately 44% of the 26,000 deaths from cirrhosis are due to ALD in the United States [1]. Alcoholic hepatitis, the clinical presentation of ALD, remains to be a common life threatening cause of liver failure, especially when it is severe. Chronic alcohol consumption has long been associated with progressive liver disease from steatosis to inflammation, development of hepatic cirrhosis, and the subsequent increased risk of hepatocellular carcinoma. Several studies have attempted to identify the molecular pathways, direct or indirect, affected by alcohol exposure in the liver. These pathways range from oxidative stress, metabolism-related effects, and inflammation to apoptosis. Induction of oxidative stress and activation of the inflammatory cascade are identified as key elements in the pathophysiology of ALD [2]. While these intracellular mechanisms affected by alcohol are studied exclusively, the interplay of signaling molecules between pathways leading to alcoholic liver disease has received less attention. Unraveling these interactions of oxidative stress mediators and inflammatory signaling in the liver will aid in identification of new integrative approaches as it relates to alcoholic liver injury and provide potential new directions to develop therapeutic target intervention. The goal of this concise paper is to first review alcohol-induced reactive oxygen species and oxidative stress generated by alcohol metabolism, endoplasmic reticulum stress, mitochondrial ROS in the liver, protein adduct formation, and autophagy and chaperone function and then to describe stress-mediated activation of receptors, kinases, and transcription factors resulting in proinflammatory signaling in ALD.

## 2. Classical Mechanisms of Alcoholic Liver Disease

Research done, so far, on the effects of pathophysiological mechanisms of alcoholic liver disease suggests the involvement of two main liver cell types, resident macrophages, or Kupffer cells and hepatocytes. The role of gut-derived endotoxin and liver macrophage activation is clearly established in ALD by Thurman and colleagues [2]. The deleterious effects of alcohol, attributed to its metabolism, primarily occur in hepatocytes [3]. Alcohol metabolism pathway including induction of cytochrome P450 2E1 [3] results in adduct formation and generation of reactive oxygen radicals respectively creating an oxidative microenvironment and damage in the liver [2]. In the currently accepted model of ALD, chronic alcohol induces oxidative stress and sensitization to endotoxin, which activates the CD14/TLR4 pathway and downstream signaling resulting in proinflammatory cytokine production [4]. The proinflammatory cytokines, particularly TNF*α*, then provoke hepatocellular injury and death by extrinsic, via TNFR1 [5] and intrinsic death pathways [6] leading to ALD. While the role of oxidative stress and macrophage activation, the two main pathophysiological processes affected in ALD, were studied independently in the past, recent studies suggest that these pathways are interconnected in ALD improving our understanding of the disease.

## 3. Reactive Oxygen Species (ROS) and Alcohol

While activation of inflammatory responses are central to alcoholic liver injury, excessive generation of reactive oxygen species plays an equally significant role in alcohol-induced cellular damage [7]. Alcohol-induced liver disease is associated with a state of “oxidative stress”. The metabolism of alcohol by alcohol dehydrogenase [ADH] leads to formation of acetaldehyde. Further, the acetaldehyde is metabolized to acetate by acetaldehyde dehydrogenase [ALDH]. Acetaldehyde, a reactive intermediate has an ability to form adducts with DNA [8, 9]. Whether acute or chronic, alcohol metabolism increases production of acetaldehyde and enhances formation of DNA adducts leading to tissue injury. On the other hand, metabolism of alcohol via cytochrome P4502E1 induces production of reactive oxygen species which facilitates adduct formation, activates stress proteins, induces endoplasmic reticulum stress, and affects lysosomal function and autophagy leading to mitochondrial injury and hepatocellular death.

### 3.1. Alcohol Metabolism and ROS

 Ethanol is primarily metabolized in the liver by oxidative enzymatic pathways. The classical pathway of alcohol metabolism involves enzymatic breakdown of alcohol by the enzyme, alcohol dehydrogenase (ADH) and its subsequent conversion to acetaldehyde and formation of acetate. ADH is predominantly expressed in liver [10] but other tissues like gastric mucosa express ADH and contribute to metabolism of alcohol [10]. Aldehyde dehydrogenase (ALDH) contributes to oxidation of aldehyde intermediates resulting in acetate which is unstable and breaks down to water and carbon dioxide. The second major pathway for ethanol degradation is the microsomal system catalyzed by cytochrome P450 enzymes. The 2E1 isoform of the cytochrome P450 (CYP2E1) system is induced during chronic alcohol consumption. Activation of CYP2E1 leads to ROS generation and highly reactive free radicals including superoxide anions and hydroxyl radicals resulting in oxidative stress and cell death [11]. The role of CYP2E1 in hepatocyte injury has been elucidated using HEPG2 cells overexpressing CYP2E1 [12], CYP2E1 knockout mice, and transgenic mice [13]. Increased oxidative stress from induction of CYP2E1 in vivo sensitizes hepatocytes to LPS and TNF*α* toxicity [14] and CYP2E1 knock-in mice showed elevated hepatic steatosis and liver injury after alcohol feeding [13]. On the other hand, CYP2E1 knockout mice showed decreased oxidant stress, upregulation of PPAR*α* and were protective to alcohol-induced liver injury. Peroxynitrite, activation of p38 and JNK MAP kinases, and mitochondrial dysfunction are downstream mediators of the CYP2E1-LPS/TNF potentiated hepatotoxicity [15]. Oxidation of ethanol by alcohol dehydrogenase and subsequent metabolism of acetaldehyde results in increased NADH/NAD+ ratio in the cytoplasm and mitochondria [16]. The increase in NADH results in inhibition of mitochondrial *β*-oxidation and accumulation of intracellular lipids [17]. Alcohol/CYP2E1-mediated ROS has the potential to peroxidize lipids and inhibit mitochondrial and peroxisomal *β*-oxidation enzymes such as acyl-CoA dehydrogenases, carnitine palmitoyl transferase-1 (CPT-1), and peroxisomal proliferator-inducing pathways, respectively [18]. This disruption leads to increased fatty acids, substrates of these enzymes, and their accumulation resulting in development of hepatic steatosis. Oxidative stress and ROS generation due to alcohol metabolism not only increase accumulation of lipids in hepatocytes but also sensitize the liver to subsequent insults by cytokines.

### 3.2. Mitochondria and Oxidative Stress

 In mitochondria, ROSs are generated as undesirable side products of the oxidative energy metabolism. An excessive and/or sustained increase in ROS production has been implicated in the pathogenesis of ALD, ischemia/reperfusion injury, and other diseases [19]. Oxidative stress induced by alcohol is closely associated with alterations in mitochondrial function resulting in cellular death. Hepatic mitochondria either acutely or chronically exposed to ethanol generate increased levels of reactive oxygen species (ROS) [20]. The induction of mitochondrial dysfunction is also linked to the metabolism of alcohol by CYP2E1 and increased oxidative stress [11]. Primary hepatocytes and rat hepatoma cells when treated with ethanol led to an increase in ROS/RNS and loss of mitochondrial function due to damaged mitochondrial DNA and ribosomes and subsequent inhibition of mitochondrial protein synthesis [21, 22]. Studies have shown that alcohol-induced ROS generation leads to alteration in mitochondrial membrane permeability and transition potential that in turn initiates the release of proapoptotic factors such as cytochrome c [21]. Transition of mitochondrial permeability results in increased caspase-3 activation in hepatocytes and this depends on p38 MAPK activation but is independent of caspase-8 [5]. Various studies show that decreased ATP synthesis accompanied by reduced mitochondrial protein synthesis, inhibition of the oxidative phosphorylation system (OxPhos), and damage to mitochondrial DNA leads to dysfunctional mitochondria and oxidative stress in alcoholic liver disease [23]. Peroxisome proliferator activated receptor gamma (PPAR*γ*)-coactivator 1 alpha (PGC-1*α*), a transcription coactivator involved in mitochondrial biogenesis, is involved in defenses against ROS by inducing many ROS-mediated detoxifying enzymes. PGC-1 gene expression was lower in hepatic tissues of rats exposed to ethanol [24]. In vitro exposure of hepatoma cells to 500 mM ethanol significantly decreased hepatic SIRT-1; PGC-1*α* leads to ROS-induced mitochondrial and cellular injury [25]. Certain sirtuins, a family of protein deacetylases, were found to regulate glucose and fat metabolism in mammals [26, 27] and to enhance mitochondrial biogenesis in liver and muscle through PGC-1*α* and to influence cell survival [28].

Recent studies used an antioxidant peptide targeted to mitochondria to show that altered ROS metabolism facilitates enhanced expression of HIF-1alpha [29], which, in turn, increases TNF-alpha secretion. These findings provide in vivo evidence for the action of mitochondrial ROS on HIF-1alpha activity and demonstrate that changes in mitochondrial function within physiologically tolerable limits can modulate the immune response [29]. These studies suggest that alcohol-induced mitochondrial stress pathways set the stage for proinflammatory cytokine-induced cell death and liver injury.

### 3.3. Protein Adducts and Lysosomal Dysfunction

 Alcohol metabolism and oxidative stress result in the formation of reactive aldehydes such as acetaldehyde, malondialdehyde (MDA), and 4-hydroxy-2-nonenal (HNE) that can bind to proteins to form adducts [30]. In vivo models of chronic alcohol consumption have shown that acetaldehyde, MDA, and HNE adduct formation are increased in various organs including the liver [30]. A strong corelation between 4-HNE adducts and expression of CYP2E1 in patients with ALD was recently shown [31]. Acetaldehyde and MDA react with proteins synergistically to form hybrid protein adducts called malondialdehyde-acetaldehyde (MAA) adducts [32]. Recognition of MAA-adducts by Kupffer cells, endothelial, and stellate cells via the scavenger receptor resulted in upregulation of cytokine and chemokine production and increased expression of adhesion molecules [32]. Circulating antibodies to MAA-adducts were detected in patients with alcoholic hepatitis and cirrhosis and correlated with the severity of liver injury [33]. Chronic alcohol feeding also induces formation of gamma-ketoaldehyde protein adducts in mouse livers [34]. These adducts are formed in a TNFR1/CYP2E1 dependent, but cyclooxygenase-independent manner in mouse liver [34]. Existence of protein adducts during chronic alcohol consumption and their identification in animal models has been challenging, limiting investigation of their precise role in ALD.

 Increased ROS and lipid peroxidation rate in microsomal and lysosomal membranes with a simultaneous decrease in the levels of glutathione sulfhydryls and glutathione-S-transferase activity was observed during alcohol exposure [35]. Elevation of cathepsin B in hepatic cytosol fractions, indicating lysosomal leakage, was reported in ethanol-fed rats [36]. Lysosomal leakage was increased in alcohol-fed mice deficient in superoxide dismutase (SOD) indicating that oxidative stress correlated with loss of lysosomal function increased hepatic fat and inflammatory cell infiltration [37]. The exact mechanisms responsible for ethanol-induced changes in lysosomal function are not clear but there is evidence of enhanced lysosomal membrane fragility, which could result from either altered lipid peroxidation, oxidative stress, or both [38]. More recently, degradation of a cell's own cytosolic components in the lysosomes as a protective mechanism against the damaging effects of oxidative stress has been described and is termed autophagy [38]. Alcoholic liver injury is associated with decreased autophagy resulting in accumulation of damaged proteins and liver cell death [38]. Recent studies show that macro-, micro- and chaperone-mediated autophagy is linked to innate and adaptive immune responses [39]. While autophagy acts as an effector and regulator of pattern recognition receptors including TLR4 signaling in macrophages, loss or defective autophagy results in accumulation of cytosolic components and chronic inflammatory responses [40]. How loss of autophagy after chronic alcohol consumption contributes to proinflammatory responses in alcoholic liver disease remains to be investigated.

### 3.4. Endoplasmic Reticulum (ER) Stress

The unfolded protein response (UPR) is a protective response of the cell also referred to as the ER stress response during pathological conditions. In alcoholic liver disease, increased expression of glucose regulatory protein (GRP)78, GRP94, CHOP, and caspase-12 indicated a UPR/ER stress response [41]. Upregulation and activation of ER-localized transcription factors such as SREBP-1c and SREBP-2 were associated with increased lipid accumulation and induction of fatty liver during chronic alcohol exposure [42]. Another important inducer of ER stress, homocysteine, was increased in alcoholic human subjects leading to hyperhomocysteinemia, also observed in alcohol feeding rodent models [43]. The role of ER stress in triglyceride accumulation and fatty liver comes from studies showing that betaine increases an enzyme, betaine homocysteine methyltransferase (BHMT) and reduces homocysteine levels to inhibit lipid accumulation [43]. Recent studies suggest that ER/UPR stress pathways intersect with innate immune signaling determining the duration and intensity of inflammatory response [44]. Additional mechanistic studies to link ER/UPR stress and innate immune responses as a pathophysiological contributor in ALD are warranted.

### 3.5. Alcohol, Stress, and Molecular Chaperones

 Stress or heat shock proteins (hsps) are ubiquitous and highly conserved proteins, functioning as molecular chaperones, whose expression is induced by oxidative stress stimuli and in response to accumulation of unfolded cellular proteins. Oxidative stress induces heat shock proteins via activation of the heat shock transcription factor (HSF) [45]. Male Wistar rats fed with acute as well as chronic alcohol showed induction of hsp70 in the various regions of the brain and the liver [46, 47]. However, the intensity of induction of hsp70 in the liver, the principal organ of ethanol oxidation, was much lower than the hippocampus or striatal areas of the brain [47]. Hsp90 levels, on the other hand, were increased in cultured rat hepatocytes exposed to acute alcohol [47, 48]. Acute and chronic alcohol treatment of monocytes/macrophages showed alterations in hsp70 and hsp90 mRNA and protein levels based on the length of alcohol exposure [49]. Acute alcohol induces HSF and hsp70, whereas chronic alcohol induces hsp90 but not hsp70 protein, through activation of HSF [49]. Hsp90 functions as a molecular chaperone controlling activity of various kinases and signaling molecules of the LPS signaling pathway such as CD14 [50], IKK [51], and IRAK [52]. Comprehensive studies on the effect of acute and chronic alcohol exposure on chaperone function of hsps in inflammatory responses in the alcoholic liver could provide novel mechanistic insights in ALD.

## 4. Inflammatory Response and ALD

Extensive studies over the past two decades have identified the importance of macrophage activation in the liver by gut-derived endotoxin after prolonged alcohol consumption [2]. Central to this activation is the sensitization of macrophages due to alcohol exposure and is associated with mechanisms ranging from upregulation and engagement of surface receptors on innate immune cells, intracellular kinases and transcription factors contributing to induction of proinflammatory cytokines.

### 4.1. Pattern Recognition Receptors, Alcohol, and Immune Cells

Pattern recognition receptors (PRRs) are expressed on liver nonparenchymal and parenchymal cells and function as sensors of microbial danger signals enabling the vertebrate host to initiate an immune response. The complexity of cellular expression of PRRs in the liver provides unique aspects to pathogen recognition and tissue damage in the liver [53]. Toll-like receptors (TLRs) that are membrane associated or endosomal recognize distinct microbial components and activate different signaling pathways by selective utilization of adaptor molecules [54]. TLRs such as TLR4 and TLR2 that detect PAMPs like LPS and lipoproteins, respectively, are located on the cell surface whereas; TLRs such as TLR3, TLR7, and TLR9 that detect viral RNA and DNA are located in the endosome [55]. The pivotal role of TLR4 as well as other TLRs has been extensively studied in alcoholic tissue injury [56–59].

The interaction of oxidative stress and TLR signaling is emerging. TLR4 is capable of inducing ROS leading to oxidative stress [59–61]. Kupffer cells or hepatic macrophages produce reactive oxygen species (ROS) in response to antigenic stimuli and chronic alcohol exposure as well as endotoxin [62, 63]. Alcohol-induced sensitization of macrophages to LPS has been attributed to ROS production [59, 60, 64]. Previous studies from Nagy and colleagues [64, 65] also show that chronic ethanol feeding increases the sensitivity of Kupffer cells to lipopolysaccharide (LPS), leading to increased tumor necrosis factor alpha (TNF*α*) expression. NADPH oxidase and ROS generation exhibit direct interaction with the TLR4 receptor and activation of down-stream kinases and transcription factors [61]. Studies by Gustot et al. [59] show that oxidative stress regulates TLR 2, 4, 6, and 9 mRNA expression in alcoholic liver. Thus, it appears that TLR mRNA, protein expression, and immune signaling can be strongly influenced by oxidative stress in ALD making these two events dependent on each other and not mutually exclusive. Besides ROS, TLRs also mediate responses to host molecules including intracellular mediators [66]. Amongst the well-characterized DAMPs, high-mobility group box 1 (HMGB1), S100 proteins, hyaluronan, and heat shock protein 60 (hsp60) are known to be recognized by TLR2 and TLR4 [66, 67]. In addition, necrotic or apoptotic cells are also recognized as DAMPs by TLRs [67]. In alcoholic liver injury, apoptotic bodies, generated due to alcohol-induced oxidative stress, could be recognized by DAMPs [68] and contribute to inflammatory responses in the liver.

Activation of TLR4 recruits IRAK-1 to the TLR4 complex via interaction with MyD88 and IRAK-4 [69]. The role of MyD88, the common TLR4 adaptor molecule, was evaluated in a mouse model of alcoholic liver injury [60]. These studies showed that MyD88 knockout mice were highly susceptible to alcohol-induced fatty liver [60]. While alcohol feeding in TLR4 deficient mice prevented activation of NADPH oxidase, alcohol-fed MyD88 deficient mice showed high NADPH oxidase activity and increased oxidative stress resulting in liver injury [60].

Increasing evidence suggests that downstream signaling components activated by TLRs as well as cytokines and chemokines produced can be regulated by oxidative stress pathways. These interactions of stress pathways leading to inflammation could contribute largely to initiation and perpetuation of alcohol-related injury in the liver. The cross-talk of stress regulated intracellular molecules with TLRs, intracellular kinases and transcription factors resulting in alterations in cytokines/chemokines in ALD are of great importance.

### 4.2. MAPKs and IKKs

LPS/TLR4-induced ROS activation [61] plays an important role in activation of downstream signaling molecules such as IRAK1/4, TRAF6 leading to activation of MAP kinases and NF*κ*B during chronic alcohol exposure [69]. Mitogen-activated protein kinase [MAPK] signaling cascade plays an essential role in several cellular processes including proliferation, differentiation, and apoptosis. Acute alcohol exposure results in activation of baseline p42/44 MAPK in hepatocytes [70] while chronic alcohol exposure causes potentiation of endotoxin-stimulated p42/44 MAPK, and p38 MAPK signaling in Kupffer cells leading to increased synthesis of TNF*α* [71, 72]. LPS stimulation of Kupffer cells in vitro exposed to chronic alcohol in vivo exhibited increased p38 activity and decreased JNK activity [71, 73]. Inhibition of p38 activation impaired alcohol-mediated stabilization of TNF*α* mRNA likely via interaction with tristetraprolin (TTP) [74]. On the other hand, ERK1/2 inhibition did not alter TNF*α* mRNA stability but affected mRNA transcription in chronic alcohol-exposed macrophages via Egr-1 binding to the promoter [75]. Whether alcohol-induced ROS plays a role in MAPK activation in ALD is not yet determined.

TLR4-induced MyD88-dependent and independent pathways lead to IKK kinase activation resulting in proinflammatory cytokine production [71]. Oxidative stress and ROS-mediated molecular chaperones such as hsp90 are shown to facilitate IKK kinase activity and downstream NF*κ*B nuclear activation [51]. Studies show that chronic alcohol-induced NF*κ*B activation in macrophages is due to increased hsp90 resulting in elevated IKK kinase activity [49]. Inhibition of hsp90 in chronic alcohol-exposed macrophages resulted in decreased IKK kinase activity and NF*κ*B binding suggesting a cross-talk between cellular stress and inflammatory pathways [49].

### 4.3. Transcription Factors in ALD

The transcription factor NF*κ*B is a ubiquitous transcription factor that can be activated by a large number of extracellular stimuli such as cytokines, chemokines, growth factors, and bacterial or viral products [76]. NF*κ*B activation triggers the induction of inflammatory genes and plays an important role in initiation and progression of alcoholic liver disease [69, 77]. While TLR-mediated activation of NF*κ*B is well established, ROS-induced activation of NF*κ*B occurs but remains poorly understood. Chronic alcohol exposure induces LPS/TLR4-mediated NF*κ*B activation in human monocytes and macrophages contributing to production of proinflammatory cytokine, TNF*α* [77]. Whether ROS mediates activation of NF*κ*B directly during ALD is unclear. TLR4-induced MyD88-independent signaling leads to activation of IKK*ε* and interferon regulatory factor 3 (IRF3) and downstream Type I IFN activation [78, 79]. Previous studies show that ROS mediates LPS-induced IRF3 activation [80]. Investigators found that IRF3 binds to the TNF*α* promoter in macrophages after chronic alcohol administration [81] and induces TNF*α* production. Whether alcohol-induced ROS mediates IRF3 induction to increase proinflammatory cytokines and liver injury needs further investigation.

 Alcohol-mediated fatty liver injury is associated with increased expression of genes regulating fatty acid synthesis and suppression of genes involved in fatty acid oxidation [82]. Transcription factors like SREBP and PPAR*α* play a pivotal role in fatty acid metabolism and rodent models as well as in vitro treatment studies with alcohol show downregulation of PPAR*α* mRNA [83]. Further, DNA-binding activity of PPAR*α* is significantly reduced resulting in decreased expression of target genes involved in fatty acid metabolism after alcohol exposure [83]. Decreased PPAR*α* activity was accompanied by increased oxidative stress in the liver resulting in increased sensitization of TNF*α*-induced liver injury [83].

Another transcription factor, STAT3, in alcoholic liver injury was recently investigated in hepatocyte-specific STAT3 knockout (H-STAT3KO) mice and macrophage/neutrophil-specific STAT3 KO (M/N-STAT3KO) and endothelial STAT3 mice [84]. Compared with wild-type mice, Kupffer cells from alcohol-fed hepatocyte-specific STAT3KO mice produced similar amounts of ROS and hepatic proinflammatory cytokines compared to control mice [85]. On the other hand, Kupffer cells from M/N-STAT3KO mice produced higher ROS and TNF*α* compared with wild-type controls. These results suggest that STAT3 in hepatocytes promotes ROS production and inflammation whereas myeloid cell STAT3 reduces ROS and hepatic inflammation during alcoholic liver injury [85]. Thus, STAT3 may regulate hepatic inflammatory cytokines via ROS production.

## 5. Stress and Immune Signaling: How Are They Linked in ALD?

**Figure 1:**
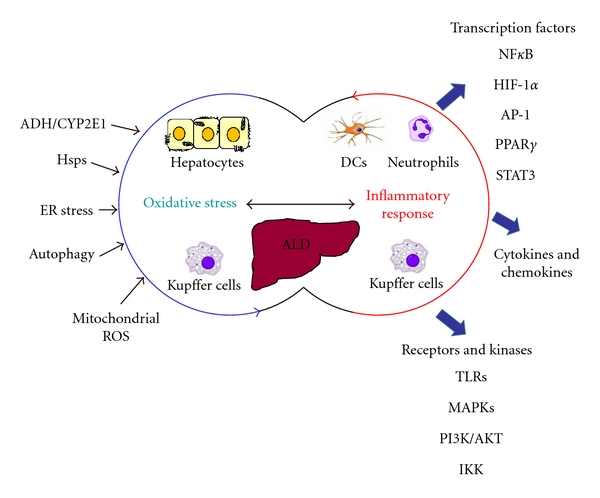
Oxidative stress and inflammation: interacting mechanisms in ALD. The development of alcoholic liver injury is a complex process involving oxidative stress microenvironment in the liver contributed by hepatocytes and macrophages. In addition to the activation of macrophages by gut-derived endotoxin, cellular stress responses contribute to proinflammatory cytokine production creating a tightly interrelated network in ALD.

Cellular stress responses during alcohol exposure include oxidative stress due to metabolism of alcohol in the liver, ER stress, mitochondrial imbalance, heat shock protein induction, and inflammatory processes. Numerous mouse models have been used to study the discrete role of each of the stress responses in alcohol-mediated liver pathology. Yet, accumulating evidence suggests that these pathways cannot be regarded separately and are tightly interrelated. Similar to other inflammatory diseases [86], alcoholic liver disease is multifactorial and it is important to take into account interactions between various cellular responses for a better understanding of the pathogenesis of ALD. Based on studies so far, a clear relationship between oxidative stress and inflammation is emerging in ALD. It is increasingly apparent that in addition to gut-derived endotoxin, alcohol-induced upregulation of oxidative stress mediators plays a major part in activation of receptors, intracellular kinases, and transcription factors in innate immune cells (Figure 1). Pathways described above that are interrelated in ALD include ROS-mediated activation of TLR4 in macrophages, mitochondrial ROS regulation of transcriptional activators such as PGC-1*α* and HIF-1*α* promoting TNF*α* induction, ROS and autophagy associated enhancement of proinflammatory cytokine production [86], ER stress-associated innate immune cell activation, hsp-mediated activation of proinflammatory signaling kinases, and finally direct activation of transcription factors such as NF*κ*B and STAT3 by ROS. Alcoholic liver disease exhibits enhanced inflammatory responses and exaggerated TNF*α* production leading to liver injury. While TNFRI knockout mice are protected from alcohol-induced liver injury [87], alcohol-induced ROS production was unaffected in TNFRI knockout mice indicating that ROS predominantly serves as a redox signal for proinflammatory cytokine production and may not be a direct toxicant to hepatocytes [87]. These studies argue against the direct role of ROS or oxidative stress in alcoholic liver injury and in fact support the notion that oxidative stress/ROS primarily affects and is indispensable to proinflammatory activation and cytokine induction in ALD creating a vicious cycle of the two pathways (Figure 1). Thus, attempts to further clarify the importance of oxidative stress and its cross-talk with inflammatory pathways will provide an insight into pathogenesis of ALD and open avenues for novel therapeutic targets.

## 6. Conclusion

 This paper clearly implicates the role of oxidative stress in proinflammatory signaling and macrophage activation during liver injury providing a feed-forward mechanism in ALD. Therefore, targeting redox-sensitive inflammatory pathways and transcription factors offers great promise for treatment of ALD. Investigation of agents that interfere with oxidative stress mediators directly hampering inflammatory cytokine production is needed. Whether these agents will then alleviate alcoholic liver disease in patients should be tested.
